# Stillbirth in COVID-19 Affected Pregnancies: A Double Whammy for the Mother

**DOI:** 10.7759/cureus.22396

**Published:** 2022-02-20

**Authors:** Sheeba Marwah, Ankita Jain, Anjali Dabral, Nitesh Gupta

**Affiliations:** 1 Department of Obstetrics and Gynaecology, Vardhman Mahavir Medical College and Safdarjung Hospital, New Delhi, IND; 2 Department of Pulmonary, Critical Care and Sleep Medicine, Vardhman Mahavir Medical College and Safdarjung Hospital, New Delhi, IND

**Keywords:** maternofetal outcomes in covid-19, stillbirth, care of covid-19 affected pregnancies, stillbirth in covid-19, adverse outcomes of sars-cov-2, covid-19 in pregnancy

## Abstract

Introduction

Pregnant women represent a high-risk group especially during the COVID-19 pandemic, suffering at the expense of pandemic restrictions and landing up in adverse maternofetal outcomes including stillbirth. Fetal demise along with COVID-19 disease acts as a double blow to these mothers. Literature is still limited on its impact on maternofetal outcomes.

Methods

A prospective, observational study was conducted in a tertiary care hospital in Delhi, India from April 15, 2020 to April 14, 2021, wherein all pregnant mothers with SARS-CoV-2 infection in the hospital who delivered a stillborn baby were enrolled and analyzed for incidence of stillbirth. These women were evaluated for risk factors and causes for stillbirth.

Results

Out of 15859 deliveries in the institute, there were 330 viable births among COVID-19 affected pregnancies. The incidence of stillbirth was 7.2% (24/330). The institutional delivery rate fell by 43% during the pandemic. The majority of cases were unbooked, from rural areas and of low socioeconomic status (p<0.01). The most significant risk factor and cause for stillbirth was an associated comorbidity (75%, p<0.001), notably severe forms of hypertensive disorders of pregnancy (HDP, 41.6%, p=0.002), followed by preterm labour (58.3%) and preterm premature rupture of membranes (PPROM, 29.1%, p<0.001). HDP remained the main cause of macerated stillbirths while maternal fever (50%, p<0.001) was the main cause of fresh stillbirth. Major modifiable factors were lack of awareness of when to seek care (83.3%), financial reasons (75%), commutation problems (87.5%), distance to hospitals (50%) and delayed referral (41.6%).

Conclusion

Improved policy-making, with an emphasis on telemedicine, COVID-19 preparedness alongside amped up vaccination and healthcare workers training will help reduce adverse maternofetal outcomes.

## Introduction

A novel Coronavirus identified as Severe Acute Respiratory Syndrome Coronavirus-2 (SARS-CoV-2) hit the world towards the end of 2019, causing a respiratory illness called coronavirus disease-19 (COVID-19). The disease evolved as an epidemic in China and then progressed to spread worldwide rapidly, declared as a pandemic on March 11, 2020 [1,2]. To date, COVID-19 has affected about 153 million people globally, with around 3.2 million deaths [3]. Likewise in India, there have been 20.2 million cases and 221,000 deaths thus far [1,3].

Owing to the unknown behaviour of SARS-CoV-2 and the immense magnitude of the COVID-19 outbreak, rigorous containment measures were adopted by governments all over the world to reduce its spread and detrimental effects on healthcare [1,4]. This included national/local lockdowns to restrict public movement to curtail the spread of the disease. This brought unprecedented changes in the landscape of medical services, access and care [5]. Subsequently, healthcare facilities saw a decrease in the outpatient attendance and admission numbers, largely due to fear of contracting the infection while seeking a doctor amidst the backdrop of imposed travel restrictions. This has been disrupting the access and delivery of healthcare services, including maternal and newborn care, especially in low-resource countries [6].

Research has shown that a reduction of 10% in mother and child healthcare coverage even in non-COVID times could result in an excess of 28,000 maternal deaths and 168,000 neonatal deaths worldwide, as was seen during Ebola virus outbreaks in 2014-16 in West Africa [7,8]. Similarly, a model-based study spanning 118 countries to gauge the access to health services during the COVID-19 outbreak reinforced the importance of continuum of care [9]. The improvement that India has shown in institutional delivery and maternal-neonatal mortality over the past decade, is at risk now [10,11]. Also, a high proportion of the rural population coupled with illiteracy has led to the fear, stigma, and superstitions related to COVID-19. There is a dearth of studies related to the impact of COVID-19 on maternal and perinatal outcomes, especially in low resource countries like India.

Stillbirth on the other hand leaves the couple destitute and with unimaginable sorrow. These bereaving parents constitute a high-risk group for potential long-lasting psychological sequelae. Battling COVID-19 physically and socially along with a fetal loss imposes grief which is unfathomable. This delicate situation calls for simultaneous empathy and adept addressal of medical concerns of SARS-CoV-2 positive mothers from the healthcare professionals.

The present study was undertaken to determine the incidence of stillbirths in COVID-19 affected pregnancies, and to ascertain the risk factors and causes of stillbirth in the same. This will guide the budding clinicians with comprehensive management of such women to avert perinatal morbidity. It will also aid in formulating standard national protocols for the same, which could be instrumental in devising preventive strategies for obtaining optimal fetal outcome.

## Materials and methods

This was a prospective, observational study conducted in the department of Obstetrics and Gynecology in a tertiary care hospital, New Delhi, India from April 15, 2020 till April 14, 2021, wherein all admitted SARS-CoV-2 positive women with intrauterine fetal demise (IUD) were enrolled in the study, after taking an informed consent from all study participants. All procedures performed in the study involving human participants were in accordance with the ethical standards of the Institutional Ethics Committee and with the 1964 Helsinki declaration and its later amendments or comparable ethical standards. Ethical clearance was obtained from Institutional Ethics Committee, VMMC and Safdarjung hospital dated 6/04/2020 reference no. IEC/VMMC/SJH/Project/2020-04/CC-04.

Any woman having an intrapartum stillbirth was also included in the study, however, women with IUD who succumbed to COVID-19 disease antenatally were excluded from the study.

For study purposes, a confirmed case of COVID-19 irrespective of symptoms was defined as a positive result on real-time reverse transcriptase-polymerase chain reaction (RT-PCR) assay of naso-oropharyngeal swab specimens or a positive rapid antigen test (RAT) [12]. Stillbirth was defined as a baby born with no signs of life after 22 weeks of gestation [13].

History was taken and examination was done to evaluate for risk factors and causes for stillbirth. The couple was appropriately counselled regarding the outcome and managed as per the institutional protocol, besides treating the cause if any. All patients were followed up till discharge.

The presentation of the categorical variables was done in the form of numbers and percentages (%). On the other hand, the quantitative data were presented as the mean ± SD and as median with 25th and 75th percentiles (interquartile range). The data normality was checked by using Kolmogorov-Smirnov test. The cases in which the data was not normal, non-parametric tests were used. The comparison of the variables which were qualitative in nature was analyzed using the Chi-Square test. If any cell had an expected value of less than 5 then Fisher’s exact test was used.

The data entry was done in the Microsoft EXCEL (Microsoft® Corp., Redmond, WA, USA) spreadsheet and the final analysis was done with the use of Statistical Package for Social Sciences (SPSS) software, version 21.0 (IBM Corp., Armonk, NY, USA). Chi-square test was applied, keeping null hypothesis value of 15% for risk factors, clinical profile, and possible modifiable factors. For statistical significance, a p-value of less than 0.05 was considered statistically significant.

## Results

During the study period, 15859 deliveries occurred in the department. There were 402 SARS-CoV-2 positive women admitted in the department and 330 viable births among them. The incidence of stillbirth was 24 out of 330 (7.2%). All of these 24 patients consented to participate in the study.

Most study participants were 21-33 years old/average being 25.5 years (p-value<0.001). The majority (75%) of the mothers belonged to rural areas and lower socioeconomic status (p-value<0.01) (Table 1).

**Table 1:** Sociodemographic and maternal characteristics of study participants n = number of participants, max. - maximum

Baseline characteristics and demographic variables	n (max. 24), (%)	Average	P-value
Age (years)		25.5	< 0.001
<30	20 (83.3)		
30-60	4 (16.6)		
Booking status			< 0.001
Unbooked	18 (75.0)		
Booked	6 (25.0)		
Residence			0.014
Rural	18 (75.0)		
Urban	6 (25.0)		
Socioeconomic status (Modified Kuppuswamy)			<0.001
Upper-middle	2 (8.3)		
Lower-middle	6 (25.0)		
Upper-lower	12 (50.0)		
Lower	4 (16.6)		
Parity		1.41	0.802
Nulliparous	8 (33.3)		
Primiparous	8 (33.3)		
Para 2-4	8 (33.3)		

The most significant risk factor for stillbirth was an associated comorbidity (75%, p<0.001). The most notable comorbidity was severe forms of hypertensive disorders in pregnancy (HDP) (41.6% cases), nine having severe preeclampsia, out of which four had HELLP syndrome (haemolysis, elevated liver enzymes and low platelets), and one patient had antepartum eclampsia (p=0.002). This was followed by preterm labour seen in nearly half of the mothers (p<0.05), while almost a third were associated with preterm premature rupture of membranes (PPROM, p<0.001). There was only one unexplained case in which no cause was found. Birth defects were not seen in any case (Tables 2, 3).

**Table 2:** Risk factors and causes for stillbirth n - number of participants, max. - maximum, PPROM - preterm premature rupture of membranes *With/without chronic hypertension

Risk factors and causes for stillbirth	n (max. 24), (%)	P-value
MATERNAL FACTORS		
Obstetric complications		
Preterm labour	14 (58.3)	<0.001
PPROM	7 (29.1)	<0.001
Chorioamnionitis	2 (8.3)	0.125
Postdatism	2 (8.3)	0.361
Associated medical comorbidity	18 (75%)	<0.001
Hypertensive disorders*	10 (41.6)	0.002
Severe preeclampsia	9	
Antepartum eclampsia	1	
Maternal anaemia (severe)	5 (20.8)	0.423
Maternal jaundice	2 (8.3)	0.124
Gestational diabetes	2 (8.3)	0.351
FETAL FACTORS		
Fetal distress	4 (16.6)	0.819
Meconium-stained liquor	2 (8.3)	0.360
UNEXPLAINED	1 (4.1)	0.783

**Table 3:** Application of the ICD-PM classification to determine causes of stillbirth n - number of participants

Causes of stillbirth	M1 Complications of placenta, cord, etc.	M2 Maternal complications of pregnancy	M3 other complications of labour & delivery	M4 Maternal medical & surgical conditions	M5 No maternal condition	Total
ANTEPARTUM DEATH						
A1: Congenital malformations, and chromosomal abnormalities	0	0	0	0	0	0
A2: Infection	0	2	0	0	0	2
A3: Antepartum hypoxia	0	0	0	12	1	13
A4: Other specified antepartum disorder	0	4	0	0	0	4
A5: Disorder related to fetal growth	0	3	0	0	0	3
A6: Antepartum death of unspecified cause	0	0	0	0	0	0
n	0	9	0	12	1	22
INTRAPARTUM DEATH						
I1: Congenital malformations, and chromosomal abnormalities	0	0	0	0	0	0
I2: Birth trauma	0	0	0	0	0	0
I3: Acute intrapartum event	0	0	0	0	2	2
I4: Infection	0	0	0	0	0	0
I5: Other specified intrapartum event	0	0	0	0	0	0
I6: Disorder related to fetal growth	0	0	0	0	0	0
I7: Intrapartum of unspecified cause	0	0	0	0	0	0
n	0	0	0	0	0	0
Total	0	9	0	12	3	24

COVID-19 symptoms were present in half of the women, such as high-grade fever (50%), followed by cough (25%) and shortness of breath (4.1%, p<0.001). These women presented at a mean gestation of 35.2 weeks with 10 delivering at term and 14 preterm deliveries. Prior history of miscarriages was noted in two (4.15%) patients; also 25% of patients had a bad obstetric history. The majority (83.3%) of patients delivered vaginally out of which half were preterm vaginal deliveries and half delivered at term. Four cases were, however, delivered by emergency caesarean section because of maternal/fetal indications. A total of 66.6% of these babies were males. Macerated stillbirth, seen in 50% of women, was observed in medical comorbidities like severe HDP, maternal anemia, and jaundice. Only two of these were seen in chorioamnionitis. On the other hand, fresh stillbirths were seen mostly in maternal infection. None of the stillbirths had congenital malformation diagnosed antenatally or noted after delivery (Table 4).

**Table 4:** Clinical profile of study participants and course in the hospital n - number of participants, max. - maximum, SB - stillbirth, BW - birth weight, kg - kilogram, ICU - intensive care unit

Clinical characteristics	n (max. 24), (%)	Average	P-value
MATERNAL PROFILE			
COVID-19 presenting symptoms			< 0.001
Asymptomatic	12 (50.0)		
Symptomatic	12 (50.0)		
High-grade fever	12		
Cough	6		
Shortness of breath	2		
Period of Gestation (weeks)		35.2	<0.001
Mode of Delivery			0.819
Vaginal	20 (83.3)		
Caesarean	4 (16.6)		
Maternal outcome			0.456
Need for ICU care	3 (12.5)		
Mortality	1 (4.1)		
STILLBIRTH (SB) DETAILS			
Time			0.732
Antepartum SB	22 (91.6)		
Intrapartum SB	2 (9.09)		
Type of SB			0.832
Fresh	12 (50.0)		
Macerated	12 (50.0)		
Birthweight (BW) (kg)		2.16	<0.001
Normal BW (2.5 to <4.0)	9 (37.5)		
Low BW (1.5 to <2.5 kg)	8 (33.3)		
Very Low BW (1 to <1.5 kg)	6 (25.0)		
Extremely Low BW (<1 kg)	1 (4.1)		
Sex			0.102
Boy	16 (66.6)		
Girl	8 (33.3)		

Major factors contributing to the ‘delay’ as per the ‘Three-Delays Model’ were: (a) delay in seeking care due to poor understanding of the complications and when to seek care (83.3%), financial reasons (75%) or lower status of women (66.6%); (b) delay in reaching care as a result of travel cost and availability (87.5%), distance to hospitals (50%) and (c) delay in receiving care as a result of delayed referral (41.6%) (Table 5).

**Table 5:** Application of three-delays model to understand modifiable factors contributing to stillbirth n - number of participants, max. - maximum

Factor	n (max. 24)	%
Delay in decision to seek care		
The low status of women	16	66.6%
Poor understanding of complications and risk factors in pregnancy and when to seek medical help	20	83.3%
Previous poor experience of health care	2	8.3%
Perceived poor quality of care at the healthcare facility	3	12.5%
Avoiding admission and long stay at a healthcare facility	8	33.3%
Financial implications	18	75%
Delay in reaching care		
Distance to health centres and hospitals	12	50%
Availability of and cost of transportation	21	87.5%
Poor roads and infrastructure	2	8.3%
Visited a traditional healer or traditional birth attendant first	3	12.5%
Delay in receiving adequate health care		
Inadequate referral systems	10	41.6%
Poor facilities and lack of medical supplies	1	4.1%

## Discussion

This was one of the earliest studies done to estimate the incidence of stillbirth in COVID-19 pregnancies. The total number of deliveries was 15859 over a period of one year in the present study, which is quite low, as compared to the delivery rate of 26,213 reported by the institute [14]. Hence, the institutional birth was reduced by 43% during the study period which is similar to a study conducted by the World Association of Perinatal Medicine (WAPM) showing a 49% decrease in institutional birth during the current pandemic [15]. This decline can be explained by more high-risk referral to our tertiary care hospital and filtering of low-risk pregnancies which got delivered at a center close to their residence, or at home, owing to fear of visiting a hospital as well as travel restrictions.

The stillbirth rate (SBR) in the pregnancies affected by SARS-CoV-2 infection was 7.2% in the present study which is way too high when compared to SBR of 2.9% in the state of Delhi and that of 2.1% reported in low-middle income countries in pre-pandemic times [16,17]. In another study by the same authors, the SBR was reported to be 2.7% in the institute before the pandemic which can be elucidated by the increase in high-risk referral to our hospital being a tertiary care institute [14]. Our findings are akin to a study in Nepal which reported almost doubling of the institutional SBR during the COVID-19 pandemic from the pre-pandemic value [11]. A recent study in the UK realized a four-fold hike in SBR (1.6%) [18]. A meta-analysis showed that there was a significant elevation in SBR during the pandemic worldwide, the most significant increases being in low-middle income countries [19]. In contrast, a Swedish study revealed no difference in SBR during the pandemic [20]. The above findings can be construed as a result of the modification in the antenatal schedule due to COVID-19 exposure, women returning for routine antenatal check-ups at longer gaps while a subset of these preferred to stay at home and reported to the hospital only during emergency. There was an amendment in obstetric care protocol warranting examination only when indicated and delay in ultrasound examination especially in pregnancies with SARS-CoV-2 positive status. Furthermore, these patients were too stressed out to keep a tab on daily fetal kick count, as a result, fetal distress was missed with around half of the mothers presenting with IUD at the time of admission to our institute.

Aforesaid observations were further augmented by the majority of patients being unbooked, hailing from rural areas and being referred. Apart from fear and initial restriction to travel, illiteracy, lack of awareness and poor adherence to antenatal schedule were quite prevalent in the study population. As is evident from past literature, this consortium of women is more prone to infection.

The main causes of stillbirth seen in the current study were preterm labour with/without PPROM and severe forms of hypertensive disorders of pregnancy (HDP). In the present study, half of the patients presented with fever at the time of admission. Literature has shown that maternal infection, fever and inflammation, all of which are at play in COVID-19 disease, leading to the release of pro-inflammatory cytokines such as interleukin-1 and tumor necrosis factor-alpha which play a role in stimulating prostaglandin production by human amnion and decidua leading to a cascade of events resulting in labour and/or rupture of membranes, and hence preterm delivery [21,22]. Furthermore, the distress of COVID-19-related social restrictions, besides psychosocial stress during pregnancy, adds to preterm delivery and birth rate [23]. Although there is still a paucity of adequate literature on this, the early studies done in Southeast Asia and the West along with a meta-analysis are consistent with increased incidence of preterm labour and delivery in COVID-19-affected pregnancies [11,15,24]. As such, the role of preterm labour as a cause for stillbirth was little if women presented to the hospital on time [14,15,24]. The adverse fetal outcomes were seen in women presenting late to the hospital, those not returning to OPD for regular check-ups, association with other medical diseases and women with acute hypoxia needing oxygen/ventilatory support.

Three-quarters of the women had an associated medical disorder of which the most significant were severe forms of pregnancy-related hypertensive disorders. This re-emphasizes the felt need that the patients came to the hospital only in case of emergency. The high incidence of HDP in COVID-19-affected pregnancies is similar to the meta-analysis done in the West, which highlights the importance of regular blood pressure (BP) monitoring [18,19,24]. Hypertension is one of the factors in the triad of maternal mortality and a big preventable cause, wherein the role of continuous home care and BP monitoring at home cannot be over-emphasized. Hospital visits were deferred due to fear and other social factors, thus, presenting to the obstetrics emergency only on the development of severe features of preeclampsia. Severe preeclampsia not only aggravates but also mimics features of COVID-19 like headache, cough and shortness of breath, which only causes further delay in diagnosis [25,26]. This highlights that preeclampsia should be picked up early for timely intervention, and in times like these, the role of telemedicine and home BP monitoring play a crucial role. It is imperative for healthcare workers to counsel all patients at the point of first contact itself regarding the importance of timely reporting to the hospital. The case-based danger signs should be explained to all pregnant women when they come to the hospital and they should also be briefed about how, when and where they can access immediate care. The policymakers need to incorporate telemedicine as a part of institutional management protocol to increase the outreach of medical care.

As per the three delays of care model, the reasons cited for the delay in seeking care were lack of awareness of the facilities that dealt with COVID-19 cases, hesitation to travel, social stigma or inability to get investigated locally at a nearby center [27]. Thus, as a result, there was delay in anticipating the complications, missing out on fetal distress and delay in management, causing grave outcomes in HDP. Obstetric triage during the pandemic is even more essential to know which patients need admission, and more importantly which don’t, considering limited hospital personnel working in staggering shifts, but at the same time not compromising with patient care or outcome [28]. Though no delay was noted at the level of healthcare institute in the present study but such delays have been quoted in recent studies [11,15,19]. Delayed referral and poor communication among various healthcare facilities need to be bolstered and augmented through nodal officers and local surveillance teams.

A fetal loss in such a situation serves as a double blow to the COVID-mother who remains isolated as per government guidelines with only tele-connect. Need of the hour becomes empathizing with the patient and, thus, the role of healthcare provider and obstetrician gets further amplified in the prevention of stillbirths, finding the cause besides treating it, helping the family acclimatize and providing guided counselling to the bereaved family.

Strengths and limitations

This is a novel study estimating the incidence of stillbirth in COVID-afflicted pregnancies in India that includes cases over a period of one year and this magnitude of deliveries. However, a drawback of our study is the limited cases obtained as it’s a single centric study. The pandemic is still continuing, further research over a period of five years and multicentric data will help in knowing the actual impact of COVID-19 on pregnancy. Another limitation is that this being a facility-based study, the stillbirths occurring at home couldn’t be assessed, hence, collaboration with a public health specialist is recommended.

## Conclusions

The incidence of stillbirth has soared in the present study owing to causes like severe HDP, preterm labour and PPROM. This necessitates early diagnosis, prompt isolation and initiation of care as per guidelines to address the risk factors for stillbirth and measures to avert intrauterine fetal hypoxia.

India is currently overcoming the second wave of COVID-19. As we step up from ‘COVID to Co-WIN' with the ongoing vaccination drive in India, COVID preparedness for prevention and management of the third wave also needs to be reinforced. Standard operating procedures (SOPs) need to be laid down for standardizing the management of high-risk pregnancies with comorbidities in the setting of a pandemic to optimize care. Besides, we need to sensitize and train healthcare providers at the grass-root level regarding the latest protocols and appropriate counseling techniques for handling COVID mothers. People globally are experiencing anxiety, stigma, lockdown and chronic mental fatigue due to COVID-19. The establishment of post-COVID-care centers for managing its long-term sequelae as well as offering mental support and bereavement care is needed. Audiovisual and mass multimedia methods need to be utilized to sensitize the general public and expecting mothers about how to access healthcare services from home. Policymaking needs to be improved and healthcare systems need to be strengthened so as to deal with both the healthcare worker safety and patient needs.
